# Does antibiotic prophylaxis for dental treatment prevent periprosthetic infections?

**DOI:** 10.3205/id000101

**Published:** 2026-01-06

**Authors:** Ursel Heudorf, Rolf Tessmann, Klaus-Peter Hunfeld

**Affiliations:** 1 Multidrug Resistant Organism (MDRO) Network Rhine-Main, Dietzenbach, Germany; 2 Institute for Laboratory Medicine, Microbiology & Infection Control, Northwest Medical Centre, Frankfurt/Main, Germany

**Keywords:** antibiotic prophylaxis (AP), antibiotic stewardship, total joint arthroplasty (TJA), periprosthetic joint infections (PJI), total hip arthroplasty (THA), total knee arthroplasty (TKA), dental procedure (DP), invasive dental procedure (IDP)

## Abstract

**Background:**

The German Society for Arthroplasty (AE) recommends a single dose of 2,000 mg amoxicillin as an antibiotic prophylaxis to prevent periprosthetic joint infections (PJI) in patients with total hip or knee arthroplasty (THA, TKA) who undergo invasive dental procedures (DP). We searched for evidence to support this recommendation.

**Materials and methods:**

We conducted a Medline query and made additional searches based on the literature found in the Medline database. We looked for relevant recommendations on antibiotic prophylaxis (AP) in other countries, as well as for standardized reviews and other studies published after the last reviews on the question of antibiotic prophylaxis for joint implant recipients in connection with dental treatment.

**Results:**

In twelve countries, no current guideline recommends general antibiotic prophylaxis for dental procedures, seven guidelines suggest that antibiotic prophylaxis should be considered in patients with risk factors, and five guidelines recommend that antibiotic prophylaxis be considered in conjunction with specific dental procedures that have an increased risk. Three reviews (2012, 2017 and 2020) mostly comprised of low-quality studies, all agreed that there is no direct evidence to indicate AP prior to dental procedures in patients with total joint arthroplasty (TJA). Six new retrospective studies from four countries on three continents, which included a total of more than 200,000 patients with TJA, confirmed the results of earlier studies: PJIs are rare and not significantly associated with DPs, and AP does not significantly reduce the (already low) risk. This applies not only to primary but also to revision TKA. Furthermore, a recent study comprising 61,124 patients with TJA or cardiac conditions who received AP for DP found that 62 (0.1%) experienced serious adverse drug events.

**Discussion:**

Even though most studies were conducted retrospectively and are based on insurance data and not on the analysis of individual medical records, it should be noted that there is still no robust evidence showing that dental procedures increase the risk of PJI, nor that AP has a risk-reducing effect both for primary THA and TKA as well as for revision TKA. Therefore, it is suggested that the AE should revise its recommendation, announced in 2022, in order to avoid the risks of unnecessary AP.

## Introduction

The German Society for Arthroplasty (Deutsche Gesellschaft für Endoprothetik, AE) recommends administering 2 g amoxicillin as an antibiotic prophylaxis (AP) for dental procedures on patients following total hip arthroplasty (THA) or total knee arthroplasty (TKA) in order to prevent late periprosthetic joint infections (PJI). In its current recommendation from January 2022 [1] the AE refers to a statement by the American Association of Orthopaedic Surgeons (AAOS) from 2009, which recommends antibiotic prophylaxis (AP) in the case of invasive dental procedures (IDP) for patients with arthroplasties [2]. The AE also refers to its own retrospective study published in 2019, in which a temporal association with a previous dental procedure (DP) was found in 7 out of 72 patients with hematogenous periprosthetic joint infections (PJI) [3]. 

The AE emphasizes the low cost of €1.30 per 1,000 mg for this prophylaxis. It also refers in this update to a large British study published just prior to the update that did not detect a statistically significant association between a previous dental procedure and periprosthetic joint infections [4]. Therefore, the AE announced in January 2022 that it would review its recommendation [1]. This revision has not yet been completed and, as a consequence, German clinics continue to recommend antibiotic prophylaxis for dental procedures and invasive dental procedures in patients with total joint arthroplasty (TJA). Against this backdrop, the Antibiotic Stewardship working group of the MDRO Network Rhein-Main, Germany, conducted a literature search on antibiotic prophylaxis for the prevention of late PJIs to find answers to the following questions:

1. What do the guidelines of other countries recommend?
2. What do the standardized reviews conclude?
3. Have newer studies been published after these reviews and what were the results?

## 1 Guidelines of various countries

**USA:** In 1997, the American Dental Association (ADA) and the American Academy of Orthopaedic Surgeons (AAOS) published their first joint advisory statement on antibiotic prophylaxis for dental procedures conducted up to 2 years after arthroplasty implantation [5]. The first revision followed in 2003 [6], which concluded that everyday dental and oral hygiene probably results in far more bacteremia from pathogens in the oral cavity than dental treatment. Neither a risk/benefit nor a cost/effectiveness analysis could justify routine antibiotic prophylaxis for the dental treatment of patients with arthroplasty, which is why it was not generally recommended. However, because limited evidence showed that certain immunocompromised patients could be at risk of a hematogenous periprosthetic joint infections, antibiotic prophylaxis could be considered for these patients during certain invasive dental procedures (tooth extractions, periodontal procedures, placement of implants, etc.) [6]. In 2009, the AAOS published an “information statement, developed as an educational tool based on the opinion of the authors.” It stated that antibiotic prophylaxis may be considered for patients with previous arthroplasty infections and for those at high risk of infection, such as immunosuppressed patients, as well as patients with co-morbidities such as diabetes, obesity, HIV, smoking, malnutrition, malignant disease, etc. [7].

Following criticism of the AAOS information statement (e.g. [7]), the ADA and AAOS published a new, joint, evidence-based guideline in 2012, replacing the previous one [8], [9]. Their recommendations were as follows:

- The practitioner might consider discontinuing the practice of routinely prescribing prophylactic antibiotics for patients with hip and knee prosthetic joint implants undergoing dental procedures. (Grade of Recommendation: Limited)
- We are unable to recommend for or against the use of topical oral antimicrobials in patients with prosthetic joint implants or other orthopaedic implants undergoing dental procedures. (Grade of Recommendation: Inconclusive)
- In the absence of reliable evidence linking poor oral health to prosthetic joint infection, it is the opinion of the work group that patients with prosthetic joint implants or other orthopaedic implants maintain appropriate oral hygiene. (Grade of Recommendation: Consensus)

In 2015, the ADA published another evidence-based clinical recommendation: “In general, for patients with prosthetic joint implants, prophylactic antibiotics are not recommended prior to dental procedures to prevent prosthetic joint infection. The practitioner and patient should consider possible clinical circumstances that may suggest the presence of a significant medical risk in providing dental care without antibiotic prophylaxis, as well as the known risks of frequent or widespread antibiotic use” [10].

In its latest recommendation from 2024, the AAOS cites four studies that found neither an association between joint implant infection and dental procedures, nor that antibiotic prophylaxis during dental procedures impacted the rate of joint implant infection. It estimates the annual cost of antibiotic prophylaxis for dental procedures of implant recipients to be 59 million US dollars, so that implementing the recommendation to withdraw from antibiotic prophylaxis use would result not only in significant cost savings for the health care system, but it would also reduce antibiotic usage and support antibiotic stewardship, thereby protecting the patients from adverse events associated with unnecessary antibiotic use [11].

**Other countries:** Looking at a compilation of guideline recommendations from 12 countries (UK, Australia, New Zealand, Canada, South Africa, France, Switzerland, Italy, Norway, Sweden, and the Netherlands), none of the guidelines recommended general antibiotic prophylaxis in patients that undergo invasive DP; 7 current guidelines suggested that antibiotic prophylaxis should be considered in patients with risk factors and 5 guidelines recommended that antibiotic prophylaxis should be considered for specific dental procedures with an increased risk [12]. A further recommendation by the Dutch Orthopedic and Dental Societies concluded: “(1) there is no indication that AP should be prescribed before dental procedures in order to prevent PJI in patients with a joint implant; (2) nor is there any indication for antibiotic prophylaxis in patients in whom an impaired immune system is supposed or confirmed; and (3) patients should be recommended to maintain good oral hygiene” [12].

**In summary,** with the exception of the above-mentioned AAOS recommendation from 2009, none of the guidelines recommend routine antibiotic prophylaxis for dental treatment in arthroplasty patients. Some of the guidelines recommend considering antibiotic prophylaxis in patients with risk factors or depending on the invasiveness of the dental procedure. Many recommendations emphasize that the supposed benefits of antibiotic prophylaxis should be weighed against the known risks of antibiotic therapy, allergies, and the development, selection and transmission of antimicrobial resistance [2], [6].

## 2 What are the conclusions of the standardized reviews?

In a comprehensive PubMed review published in 2012 on antibiotic prophylaxis in dental surgery to reduce the risk of prosthesis infection, the authors examined the following questions: Frequency and intensity of bacteremia of orodental origin, frequency of prosthesis infections due to dental surgery, and objective efficacy of antibiotic prophylaxis in dental surgery in patients with joint implants [13].

- Frequency of bacteremia of orodental origin: Short-term bacteremia occurs in 100% of cases after tooth extractions and in 17–51% of cases after standard tooth cleaning and brushing. The cumulative duration of bacteremia after dental care was estimated at 5,370 minutes per month; in the case of a tooth extraction it was 6–30 minutes. Accordingly, spontaneous bacteremia occurs much more frequently in conjunction with poor dental hygiene than after tooth extraction.
- Frequency of prosthesis infections following dental procedures: Several studies have shown that prosthesis infections following dental treatment are very rare, even without antibiotic prophylaxis; the risk after dental procedures is lower than after skin injuries. Various retrospective and one prospective study were unable to find a link between dental treatment and arthroplasty infections.
- Effectiveness of antibiotic prophylaxis in dental surgery in patients with joint implants: In a single-center case study conducted between 2001 and 2006 with 339 cases and 339 controls, the risk of arthroplasty infection was neither influenced by previous dental treatment (OR 0.8; 95% CI 0.4–1.6) nor by antibiotic prophylaxis during tooth extraction (OR 0.9; 95% CI 0.7–2.2) [14].

Thus, there was no reliable evidence that antibiotic prophylaxis of orodental surgery provides effective protection in joint implant recipients – regardless of the immune status – nor evidence of any harm caused by not administering antibiotic prophylaxis.

Further reviews were published in 2017 [12] and 2020 [15]; however, only one more recent study [16] could be included. Kao et al. conducted a retrospective, population-based cohort study with data from the Taiwan National Health Insurance Research Database (NHIRD). All Taiwanese residents (N=255,568) who underwent total knee or hip arthroplasty between January 1, 1997, and November 30, 2009, were included in the study. This total group also encompassed a subgroup of 57,066 patients who received dental treatment (dental cohort). This subgroup was individually matched 1:1 with a nondental cohort by age, sex, propensity score, and index date. The dental cohort was further divided into antibiotic and nonantibiotic subcohorts comprising 6,513 matched pairs. Periprosthetic joint infections occurred in 328 patients (0.57%) in the dental subcohort and in 348 patients (0.61%) in the nondental subcohort (HR_adj_ 0.94 (P95% CI 0.80–1.10). Furthermore, periprosthetic joint infections occurred in 13 patients (0.2%) in the antibiotic subcohort and in 12 patients (0.18%) in the nonantibiotic subcohorts (HR_adj_ 1.03 (P95% CI 0.47–2.27). The authors concluded that the risk of PJI is not increased following dental procedure in patients with hip or knee replacement and is unaffected by antibiotic prophylaxis [16].

Thus, the following was concluded in 2020: “The current systematic review, mostly composed of low-quality studies, suggests that there is no direct evidence to indicate prophylactic antibiotics prior to dental procedures in patients with total joint arthroplasty. In line with the current guidelines, no prophylaxis should be used on interventions for non-infected causes, except for occasional unusual situations, which can then be judged individually” [15].

### Further studies published after the 2020 review

Further studies have since been published. Table 1 (Tab. 1) lists the main patient data, methods, results and the authors’ conclusions.

Thornhill et al. published a study in 2022 with data from 9,427 patients (2,385 patients with hip arthroplasties, 3,168 with knee arthroplasties, 259 with other arthroplasties and 3,615 with unknown prosthesis types) who were admitted to hospitals in the UK between December 2011 and March 2017 with a hematogenous arthroplasty infection and for whom data on dental treatments were also available. Although the authors found bacteria from the oral cavity to be a possible cause in 9% of the prosthesis infections, there was no positive temporal association between invasive dental treatment and prosthesis infection. On the contrary, the incidence of invasive dental treatment was lower in the last 3 months before PJI (IRR 0.89, 95% CI 0.082–0.096; p=0.002) than in the previous 12 months. The authors suspected that the few arthroplasty infections with oral streptococci were instead the result of dental hygiene routines and they considered patients with poor oral hygiene to be at particular risk [4]. The authors therefore saw no evidence for antibiotic prophylaxis during invasive dental treatment to prevent hematogenous prosthesis infections.

Thornhill et al. presented a further study in 2023 that now included 2,344 patients in the USA who were hospitalized due to a late infection of a hip prosthesis. These patients had undergone a total of 4,614 dental procedures in the previous 15 months, including 1,821 invasive procedures, 18.3% of which involved perioperative antibiotic prophylaxis. The authors discovered that there was neither a significant association between dental treatment and endoprosthesis infection nor a significant reduction in the risk of prosthesis infection as a result of antibiotic prophylaxis [17].

Sax et al. [18] conducted a case-control study using national insurance data from 1,952,917 patients in the USA who had received primary or revision total knee arthroplasty between 2010 and 2020. The cohorts were established based on demographic and health metrics: a dental procedure cohort with antibiotic prophylaxis, a dental procedure cohort without antibiotic prophylaxis, and a control cohort without dental procedures. Each cohort included 496 patients. The rate of periprosthetic joint infections did not vary significantly in any of the three cohorts. Antibiotic prophylaxis and no antibiotic prophylaxis showed statistically similar odds for periprosthetic joint infections and revision at all time points (PJI: OR, 0.62; 95% CI, 0.11–4.00; P≥0.479; revision: OR, ≥0.33; 95% CI, 0.03–4.00; P≥0.248). 

Another retrospective study in the USA examined 10,894 knee or hip implants performed in one specialist clinic between January 2019 and December 2020 [19]. Some of the surgeons recommended antibiotic prophylaxis (2,000 mg amoxicillin 30 min before surgery) for invasive dental procedures, while others did not. In the study, 2,871 patients (26.4%) were in the “no antibiotic” group and 8,023 (73.6%) in the antibiotic group. A total of 27 (0.3%) late infections were identified: 24 (0.3% of 8,023) in the antibiotic group and 3 (0.1% of 2,871) in the “no antibiotic” group. A total of 4 dental-related infections, defined as infections associated with dental problems or following dental treatment, were identified, all of which occurred in the antibiotic group. The authors concluded that, based on the data, there is no need for routine antibiotic prophylaxis for invasive dental procedures following knee or hip arthroplasty [19].

Park et al. [20] used the Health Insurance Review and Assessment Service data in South Korea for their nationwide, retrospective, comparative, large-database study encompassing 591,602 patients with unilateral primary or revision total knee arthroplasty between 2009 and 2019. Here, 90% of the patients underwent a dental procedure at least 1 year after the index surgery. After propensity score matching, patients were classified into a nondental (n=61,422) and a dental cohort (n=182,052), the latter being subdivided into two groups: the AP group (66,303 patients with prophylactic antibiotics) and the non-AP group (115,749 patients without prophylactic antibiotics). The study found that dental procedures were not associated with an increase in periprosthetic joint infections risk after primary (HR_adj_ 1.56, 95% CI 0.30–8.15) or revision total knee arthroplasty (adjusted HR 1.74, 95% CI 0.90 to 3.34). Additionally, AP was not associated with a reduced PJI risk after the index surgery (primary TKA: HR_adj_ 1.28, 95% CI 0.30–5.42; revision TKA: HR_ad__j_ 0.74, 95% CI 0.45–1.23). The authors concluded that, based on their findings, there is insufficient rationale for administering prophylactic antibiotics before dental procedures in patients who have undergone primary or revision total knee arthroplasty [20]. 

A recent study in Japan was conducted using a commercially available administrative claims database provided by DeSC Healthcare that included approximately 12 million inhabitants. The authors conducted a case-crossover study with patients who had undergone dental procedures and were hospitalized for late periprosthetic joint infections (LPJI) between April 2014 and September 2021. A total of 241 patients with LPJI were included in the case-crossover study. Cases were defined as exposure to DP 1–4 weeks prior to an LPJI hospital admission. Exposure to dental procedures in the controls was divided into two control periods of 9–12 weeks and 17–20 weeks prior to an LPJI hospital admission. The OR for LPJI with dental procedures was 0.96 (95% CI, 0.61–1.53). Stratification by AP during the dental procedure included antibiotic prophylaxis and did not change the results [21].

Finally, in 2024, a comprehensive paper was published on the adverse effects of antibiotic prophylaxis during dental treatment in patients with heart disease or joint prostheses to prevent hematogenous prosthesis infection or endocarditis [22]. Of the 61,124 patients who received antibiotic prophylaxis for dental interventions between 2015 and 2017, 62 (0.1%) experienced serious adverse drug events (ADEs), including 42 allergic reactions, 1 anaphylactic shock, and 19 Clostridioides difficile infections. Here, 18 (0.09%) ADEs occurred after guideline-compliant indication of AB prophylaxis and 44 (0.1%) after non-guideline-compliant antibiotic prophylaxis.

As a result, all studies published after the last review in 2020 confirmed the results of earlier studies. Periprosthetic joint infections were rare and were not significantly associated with PDs [4], [17], [19], [20]. An AP did not significantly reduce the (low) risk [17], [18], [19], [20], [21]. This applies not only to primary but also to revision TKA [20]. Thus, there was no benefit in applying antibiotic prophylaxis before DP in patients with joint implants. On the contrary, Thornhill et al. [17] concluded that the continued use of AP posed an unnecessary risk to patients due to adverse drug reactions, and to society from the potential of antibiotic prophylaxis to promote the development of antibiotic resistance. Though small, the risk for serious adverse drug events (ADEs) due to non-evidence-based antibiotic prophylaxis must not be overlooked. 

## Limitations

The reviews cited here emphasized that they were mostly only based on low-quality studies. In general, big-data or real-world-data studies, in which secondary data is evaluated retrospectively, are inferior to high-quality randomized studies and are not a substitute for them [23]. This also applies to the studies cited here. They did include large numbers of patients, however, they referred to secondary data that were collected for reimbursement purposes (health insurance data) or for statistical purposes. Furthermore, the studies were conducted retrospectively with these datasets. It should be noted that the quality and completeness of such data is less reliable than in studies conducted prospectively with a study team. In addition, it was not always certain whether the antibiotic prophylaxis was only prescribed but not taken. At the same time, it cannot be ruled out that patients could have received antibiotics for other diagnoses. However, on a positive note, the studies included large and, with one exception [19], multicenter patient collectives from various countries and continents. Different and, in some cases, interesting study designs were used, including case-crossover and time-trend approaches, as well as case control with patient groups matched according to sociodemographic data and risk factors.

## Conclusion

With the exception of the AAOS information statement from 2009, which was withdrawn in 2012, none of the cited guidelines recommend general antibiotic prophylaxis in line with the reviews published up to 2020 and the studies published since then and referred to here. Even though most studies were conducted retrospectively and are based on insurance data and not on the analysis of individual medical records, it should be noted that there is still no robust evidence that dental procedures increase the risk of PJI, nor that antibiotic prophylaxis has a risk-reducing effect, both for primary total hip arthroplasty and total knee arthroplasty as well as for revision total knee arthroplasty. Therefore, we suggest that statements as issued by some scientific societies [1] should be revised in order to avoid the risks of unnecessary antibiotic prophylaxis.

## Notes

### Competing interests

The authors declare that they have no competing interests.

## Figures and Tables

**Table 1:**
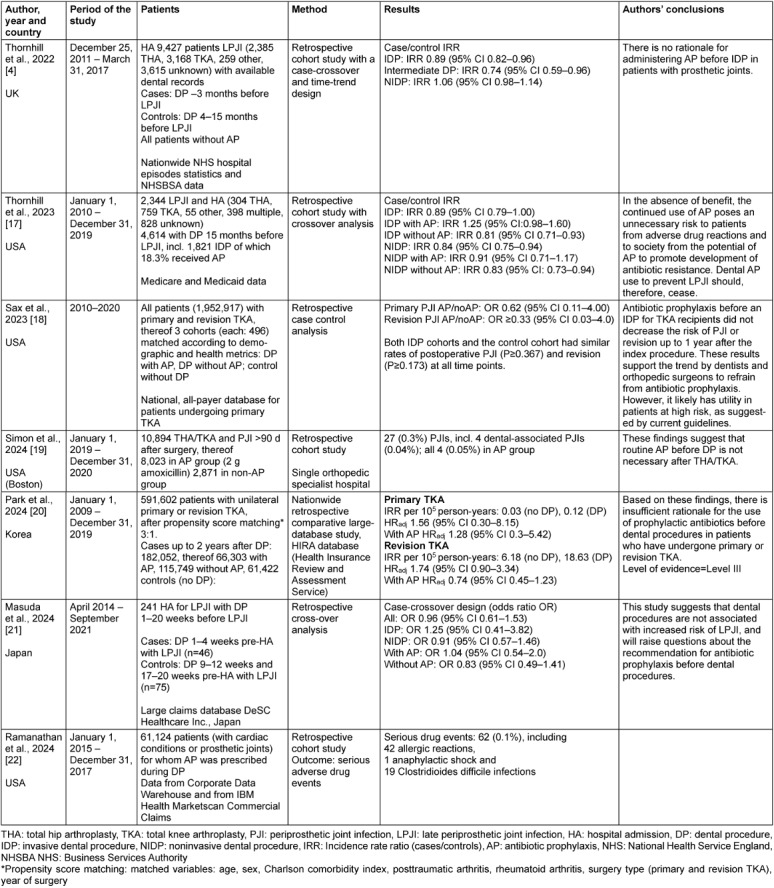
Studies published after the 2020 review

## References

[1] AE – Deutsche Gesellschaft für Endoprothetik Hüft- und Knieprothese: Implantatinfekte durch blutige Zahn-OPs? Neue Studie hält Antibiotikaprophylaxe für überflüssig.

[2] American Academy of Orthopaedic Surgeons (AAOS) (2009). Information statement: antibiotic prophylaxis for bacteremia in patients with joint replacements.

[3] Rakow A, Perka C, Trampuz A, Renz N (2019). Origin and characteristics of haematogenous periprosthetic joint infection. Clin Microbiol Infect.

[4] Thornhill MH, Crum A, Rex S, Stone T, Campbell R, Bradburn M, Fibisan V, Lockhart PB, Springer B, Baddour LM, Nicholl J (2022). Analysis of Prosthetic Joint Infections Following Invasive Dental Procedures in England. JAMA Netw Open.

[5] American Dental Association, American Academy of Orthopaedic Surgeons (1997). Antibiotic prophylaxis for dental patients with total joint replacements. American Dental Association; American Academy of Orthopaedic Surgeons. J Kans Dent Assoc.

[6] American Dental Association, American Academy of Orthopaedic Surgeons (2003). Antibiotic prophylaxis for dental patients with total joint replacements. J Am Dent Assoc.

[7] Napeñas JJ, Lockhart PB, Epstein JB (2009). Comment on the 2009 American Academy of Orthopaedic Surgeons' information statement on antibiotic prophylaxis for bacteremia in patients with joint replacements. J Can Dent Assoc.

[8] Rethman MP, Watters W 3rd, Abt E, Anderson PA, Carroll KC, Evans RP, Futrell HC, Garvin K, Glenn SO, Goldberg MJ, Hellstein J, Hewlett A, Kolessar D, Moucha C, O' Donnell RJ, Osmon DR, O' Toole J, Rinella A, Steinberg MJ, Martin WR 3rd, Cummins DS, Song S, Sluka P, Boyer K, Woznica A, Ristic H, Hanson NB, American Academy of Orthopaedic Surgeons, American Dental Association (2013). The American Academy of Orthopaedic Surgeons and the American Dental Association clinical practice guideline on the prevention of orthopaedic implant infection in patients undergoing dental procedures. J Bone Joint Surg Am.

[9] American Academy of Orthopaedic Surgeons, American Dental Association (2012). Prevention of Orthopaedic Implant Infections in Patients Undergoing Dental Procedures. Evidence-Based Clinical Practice Guideline.

[10] Sollecito TP, Abt E, Lockhart PB, Truelove E, Paumier TM, Tracy SL, Tampi M, Beltrán-Aguilar ED, Frantsve-Hawley J (2015). The use of prophylactic antibiotics prior to dental procedures in patients with prosthetic joints: Evidence-based clinical practice guideline for dental practitioners--a report of the American Dental Association Council on Scientific Affairs. J Am Dent Assoc.

[11] American Academy of Orthopaedic Surgeons, American Association of Hip and Knee Surgeons (2024). The Prevention of Total Hip and Knee Arthroplasty Periprosthetic Joint Infection in Patients Undergoing Dental Procedures. Evidence-Based Clinical Practice Guideline.

[12] Rademacher WMH, Walenkamp GHIM, Moojen DJF, Hendriks JGE, Goedendorp TA, Rozema FR (2017). Antibiotic prophylaxis is not indicated prior to dental procedures for prevention of periprosthetic joint infections. Acta Orthop.

[13] Legout L, Beltrand E, Migaud H, Senneville E (2012). Antibiotic prophylaxis to reduce the risk of joint implant contamination during dental surgery seems unnecessary. Orthop Traumatol Surg Res.

[14] Berbari EF, Osmon DR, Carr A, Hanssen AD, Baddour LM, Greene D, Kupp LI, Baughan LW, Harmsen WS, Mandrekar JN, Therneau TM, Steckelberg JM, Virk A, Wilson WR (2010). Dental procedures as risk factors for prosthetic hip or knee infection: a hospital-based prospective case-control study. Clin Infect Dis.

[15] Slullitel PA, Oñativia JI, Piuzzi NS, Higuera-Rueda C, Parvizi J, Buttaro MA (2020). Is there a Role for Antibiotic Prophylaxis Prior to Dental Procedures in Patients with Total Joint Arthroplasty? A Systematic Review of the Literature. J Bone Jt Infect.

[16] Kao FC, Hsu YC, Chen WH, Lin JN, Lo YY, Tu YK (2017). Prosthetic Joint Infection Following Invasive Dental Procedures and Antibiotic Prophylaxis in Patients With Hip or Knee Arthroplasty. Infect Control Hosp Epidemiol.

[17] Thornhill MH, Gibson TB, Pack C, Rosario BL, Bloemers S, Lockhart PB, Springer B, Baddour LM (2023). Quantifying the risk of prosthetic joint infections after invasive dental procedures and the effect of antibiotic prophylaxis. J Am Dent Assoc.

[18] Sax OC, Bains SS, Chen Z, Delanois RE, Nace J (2023). Antibiotic Prophylaxis Is Not Necessary for Invasive Dental Procedures in Existing Total Knee Arthroplasty Implants. Orthopedics.

[19] Simon SJ, Aziz AA, Coden GS, Smith EL, Hollenbeck BL (2024). Antibiotic Prophylaxis Prior to Dental Procedures After Total Hip and Knee Arthroplasty Does Not Decrease the Risk of Periprosthetic Joint Infection. J Arthroplasty.

[20] Park HJ, Koh K, Choi YJ, Suh DH, D'Lima D, Kim JG (2024). Is Prophylactic Antibiotic Use Necessary Before Dental Procedures in Primary and Revision TKA? A Propensity Score-matched, Large-database Study. Clin Orthop Relat Res.

[21] Masuda S, Fukasawa T, Takeuchi M, Arai K, Matsuda S, Kawakami K (2024). Association between dental procedures and periprosthetic joint infection: A case-crossover study. J Orthop Sci.

[22] Ramanathan S, Evans CT, Hershow RC, Calip GS, Rowan S, Hubbard C, Suda KJ (2024). Guideline concordance and antibiotic-associated adverse events between Veterans administration and non-Veterans administration dental settings: a retrospective cohort study. Front Pharmacol.

[23] Windeler J, Lauterberg J, Wieseler B, Sauerland S, Lange S (2017). Patientenregister für die Nutzenbewertung. Kein Ersatz für randomisierte Studien. Deutsches Ärzteblatt.

